# Oncogenic epithelial cell-derived exosomes containing Rac1 and PAK2 induce angiogenesis in recipient endothelial cells

**DOI:** 10.18632/oncotarget.7573

**Published:** 2016-02-22

**Authors:** Shashi K. Gopal, David W. Greening, Eric G. Hanssen, Hong-Jian Zhu, Richard J. Simpson, Rommel A. Mathias

**Affiliations:** ^1^ Department of Biochemistry and Genetics, La Trobe Institute for Molecular Science, La Trobe University, Melbourne, Victoria, Australia; ^2^ Bio21 Institute, The University of Melbourne, Melbourne, Victoria, Australia; ^3^ Department of Surgery, The University of Melbourne, Melbourne, Victoria, Australia

**Keywords:** epithelial-mesenchymal transition, exosome, angiogenesis, tumour microenvironment, extracellular vesicle

## Abstract

The metastatic cascade describes the escape of primary tumour cells to distant secondary sites. Cells at the leading tumour edge are thought to undergo epithelial-mesenchymal transition (EMT), to enhance their motility and invasion for spreading. Whether EMT cells directly promote tumour angiogenesis, and the role of exosomes (30-150 nm extracellular vesicles) remains largely unknown. We examined the functional effects of exosomes from MDCK cells, MDCK cells stably expressing YBX1 (MDCK^YBX1^, intermediate EMT), and Ras-transformed MDCK cells (21D1 cells, complete EMT). 2F-2B cell motility and tube formation (length and branching) was significantly increased following supplementation with MDCK^YBX1^ or 21D1 exosomes, but not MDCK exosomes. Next, Matrigel™ plugs containing exosome-supplemented 2F-2B cells were subcutaneously injected into mice. Systemic perfusion was only observed for plugs supplemented with MDCK^YBX1^ or 21D1 exosomes. Comparative proteomics revealed that 21D1 exosomes contained VEGF-associated proteins, while MDCK^YBX1^ exosomes were enriched with activated Rac1 and PAK2. To validate, 2F-2B cells and HUVECs were pre-treated with PAK inhibitors prior to exosome supplementation. PAK inhibition nullified the effects of MDCK^YBX1^ exosomes by reducing the tube length and branching to baseline levels. By contrast, the effects of 21D1 exosomes were not significantly decreased. Our results demonstrate for the first time that oncogenic cells undergoing EMT can communicate with endothelial cells via exosomes, and establish exosomal Rac1/PAK2 as angiogenic promoters that may function from early stages of the metastatic cascade.

## INTRODUCTION

Interactions occurring between tumour cells at the invasive front and cells in the tumour microenvironment (TM) promote cancer invasion and metastasis [1-4]. Cells at the leading tumour edge are thought to undergo epithelial-mesenchymal transition (EMT) in response to a variety of growth factors, signalling molecules and transcription factors [5]. EMT results in heightened cell motility and invasiveness through diminished cell–cell and cell–matrix adhesion, reorganisation of the cytoskeleton, and remodelling of the ECM [6]. Importantly, EMT is involved in the process of cancer cell intravasation into blood and/or lymph vessels [6, 7]. However, the contribution of EMT cells to the recruitment of endothelial cells and the formation of blood vessels remains not well understood.

Tumour angiogenesis and vessel formation can be controlled by signals in the TM that permit vascular remodelling via a series of controlled events [8, 9]. Stimulation and activation of endothelial cells drives their detachment from junctional adhesions, sprouting, and migration and proliferation, forming provisional tubes and functional networks [8]. Secreted molecules have the ability to influence endothelial cell behaviour [10]. For example, growth factors (VEGF, PDGF, FGF), signalling molecules (TGF-β, Notch, Wnt, ANG and TIE), integrins (α_v_β_3_ and α_v_β_5)_ and proteases (membrane type 1-MMP and MMP9) are known to regulate endothelial cell functions and neovascularisation [11]. Given that extracellular vesicles (EV) have recently been reported as modulators of the TM [12, 13], we speculate that they may also promote angiogenesis.

Cancer cells can communicate with recipient endothelial cells through nano-sized 30-150 nm EVs known as exosomes [14-18]. Exosomes from leukemic and glioblastoma cells have been shown to increase endothelial cell tube formation and neovascularisation in *in vivo* matrigel plugs [14, 15, 18]. Micro-RNA, miR-92a contained in leukaemia-derived exosomes stimulated endothelial cell migration and tube formation[16]. Despite the identification of these molecular effectors beginning to emerge, precisely when tumour angiogenesis is initiated, in the context of the metastatic cascade, remains to be defined. Moreover, the ability of EMT cells to promote tumour angiogenesis has not yet been investigated.

We have previously shown that constitutive expression of H-Ras in MDCK cells (21D1 cells) induces all the phenotypic hallmarks of EMT, and characterized alterations to the secretome, plasma membrane, and exosome protein profiles [19-22]. More recently, we have been interested in defining the earlier events that may give rise to the partial EMT (p-EMT) phenotype. Stable expression of the pleiotropic transcription/splicing factor and RNA-binding protein, nuclease-sensitive element-binding protein 1 (YBX1/YB-1), increased the oncogenicity of MDCK cells (MDCK^YBX1^) and increased secretion of soluble-secreted proteins associated with promoting angiogenesis [23]. In the present study, we investigated the downstream functional consequences of treating recipient endothelial cells with exosomes derived from MDCK, MDCK^YBX1^, and 21D1 cells. We discovered that as oncogenicity increases (MDCK^YBX1^ < 21D1 cells), so does the potency of the cell-derived exosomes to induce angiogenesis in recipient endothelial cells. Nonetheless, exosomes derived from MDCK^YBX1^ cells induced a pronounced angiogenic response, and this suggests that tumour angiogenesis may commence during early stages of the metastatic cascade, such as by p-EMT cells.

## RESULTS

We have previously observed that over-expression of YBX1 in MDCK cells induces p-EMT, and causes elevated release of soluble secreted proteins (TGF-β, CSF-1, NGF, VGF, ADAM9 and ADAM17) associated with promoting angiogenesis [23]. In this current study, we focussed on the functional contribution exosomes derived from increasingly oncogenic EMT cells (MDCK < MDCK^YBX1^ < 21D1) may have on inducing angiogenesis in recipient endothelial cells.

### Isolation and characterisation of extracellular vesicles

**Figure 1:**
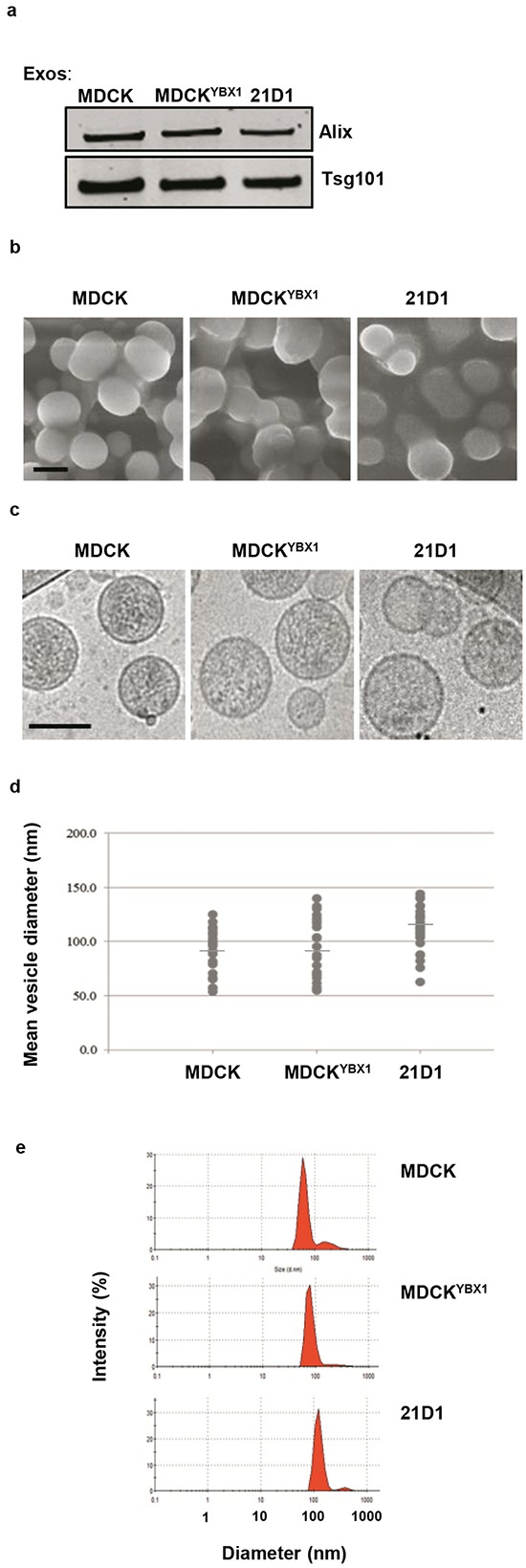
Isolation and characterisation of exosomes from EMT cell lines **a.** Exosomes were isolated, purified and examined for expression of exosome markers Alix and TSG101 by western immuno-blotting. **b.** Vesicle morphology assessed by scanning electron microscopy. Representative image from n=3 and 5 independent fields of view. Scale bar = 100nm. **c.** Analysis of exosomes by cryo-electron microscopy. Representative image from n=3 and 5 independent fields of view. Scale bar = 100nm **d.** Distribution of exosome diameter by cryo-electron microscopy (average from *n*=3, 5 independent fields of view). **e.** Exosome diameter assessed by dynamic light scattering (n=3).

EVs were isolated from MDCK, MDCK^YBX1^ and 21D1 cells using established workflows (Supplementary Figure S1) based on OptiPrep™ density gradient ultracentrifugation [22, 24]. Western blotting analysis showed Fraction 7, corresponding to a density of 1.09 g/mL, to have the greatest expression of exosome markers (Supplementary Figure S2), and was selected for further characterization. Fraction 7 vesicles from all cell lines showed robust expression of ESCRT machinery proteins Alix and TSG101 (Figure 1a), and scanning electron microscopy revealed spherical architecture with textured surfaces (Figure 1b). Additionally, cryo-electron microscopy and cross sectional analysis displayed densely-staining vesicular contents (Figure 1c), while size distribution indicated a homogenous population of vesicles ranging between 50-140 nm (Figure 1d). Additionally, dynamic light scattering indicated a slightly increasing mean vesicle diameter measuring 84.2nm (MDCK), 95.5 nm (MDCK^YBX1^) and (108.5 nm) (21D1) (Figure 1e). Based on these observed characteristics, Fraction 7 vesicles were classified as exosomes and used in downstream experiments.

### Exosomes can be internalized by recipient endothelial cells

**Figure 2:**
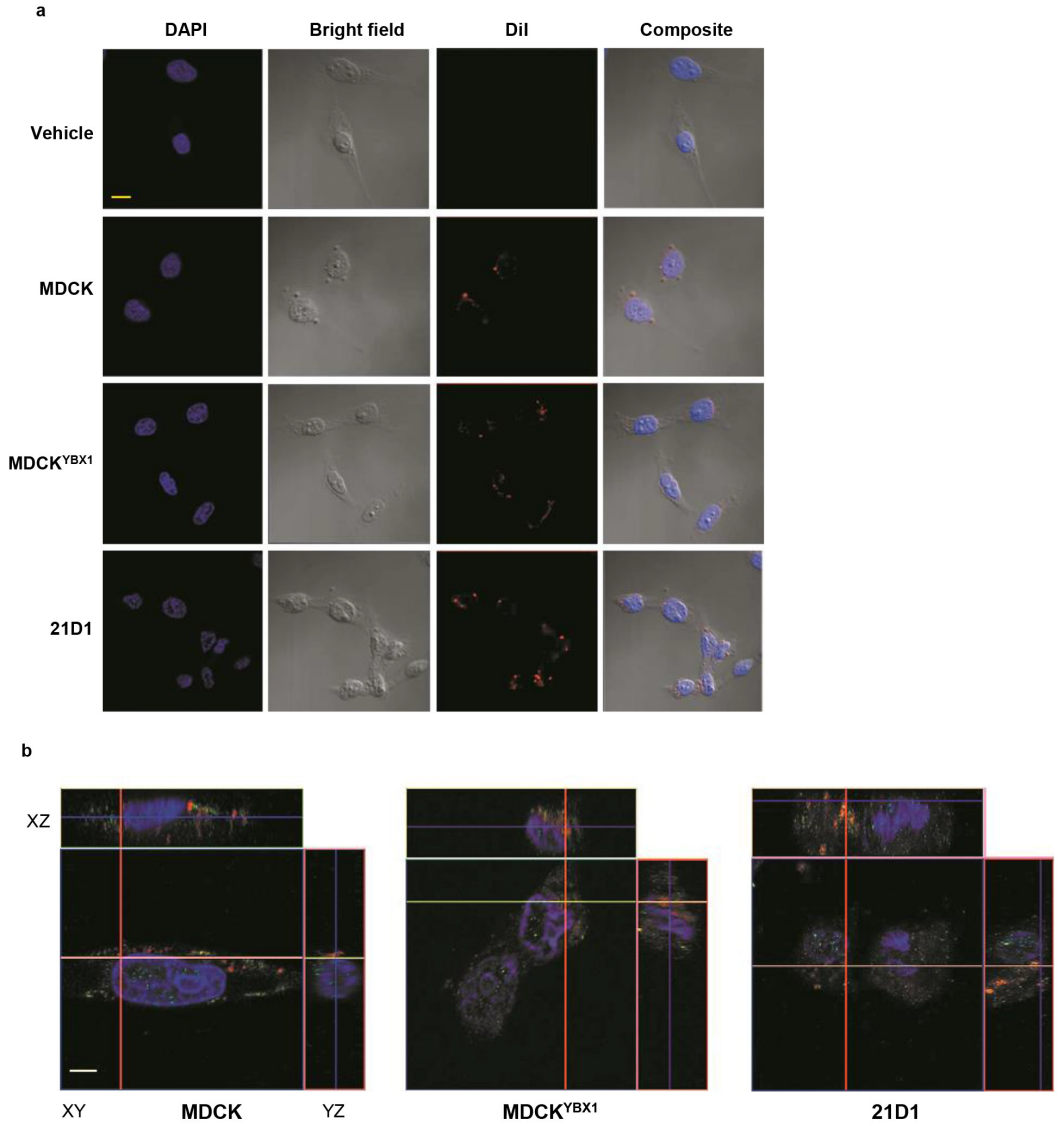
EMT cell exosomes are internalised by recipient endothelial cells **a.** 10 μg of exosomes were isolated and labelled with 1 μM DiI (red). Exosomes, or vehicle (DMEM) were supplemented to 2F-2B cells for 2 h, and stained with DAPI (blue) prior to confocal and bright-field microscopy. Scale bar = 10 μm (representative images from n=3). **b.** Following supplementation of labelled exosomes with DiI (red) for 2 h, 2F-2B cells were fixed, permeabilized and stained with DAPI (blue), and anti-actin antibodies (green). Z-stack images of cells were generated by confocal microscopy. Scale bar = 5 μm (*n*=3).

The biological function of exosomes as delivery vehicles is underpinned by their ability to deliver cargo into recipient cells. To establish whether exosomes from all three EMT model cell lines could be internalized by endothelial cells, we monitored uptake in 2F-2B cells using confocal microscopy. Exosomes were labelled with the lipophilic cationic indocarbocyanine dye DiI, and supplemented to 2F-2B cells. Following a 2 hr incubation, confocal imaging was performed and demonstrated association of the fluorescent dye with the cells (Figure 2a). Furthermore, Z-stack analysis verified that the fluorescence signal does indeed emanate from within the 2F-2B cells, and not only from extracellular membrane interaction (Figure 2b).

### Exosomes from oncogenic MDCK^YBX1^ and 21D1 cells promote 2F-2B cell angiogenesis

**Figure 3:**
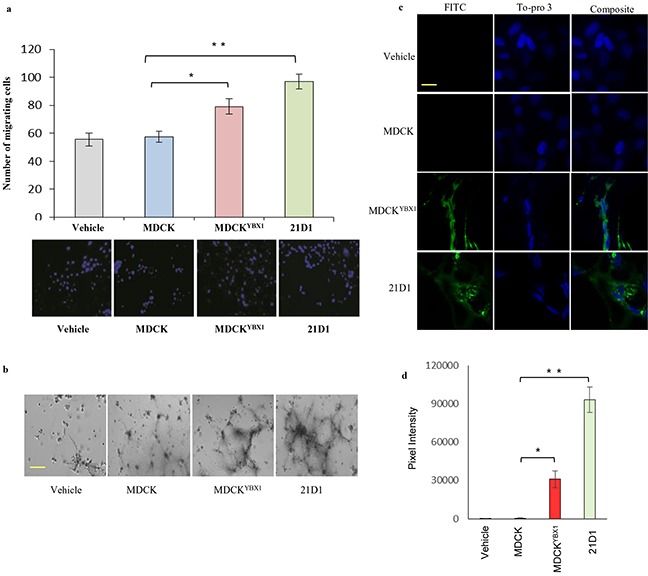
Exosomes from oncogenic cells promote endothelial cell angiogenesis *in vitro* and *in vivo* **a.** Transwell assay was used to measure 2F-2B cell migration following supplementation with exosomes (30 μg) from MDCK, MDCK^YBX1^ or 21D1 cells. After 24 h, migrating cells that passed through the transwell were stained with DAPI (blue), imaged, and counted. Scale bar = 100 μm. (*n*=3; average ± SEM, *p<0.05, **p<0.01). **b.** Tube formation assays were conducted using 1 mg/mL Matrigel. 2F-2B cells were seeded on Matrigel and supplemented with exosomes (30 μg). After 24 h of culture, tube formation was analysed and imaged. Scale bar = 50 μm (representative images from *n*=3). **c.** *In vivo* analysis of Matrigel plugs containing 2F-2B cells (2.5×10^5^) supplemented with exosomes (30 μg). Plugs were subcutaneously injected into NOD/SCID mice in both inguinal regions. After 21 days, IP tail vein injections of FITC-dextran were performed and animals sacrificed after 1 h. Matrigel plugs were sectioned (12 μm), and imaged by confocal microscopy to reveal FITC-dextran (green) and To-pro 3 (blue) expression. Scale bar = 10 μm. **d.** Quantitative analysis of FITC-dextran expression in exosome treated matrigel plugs. Pixel intensities of FITC–dextran fluorescence (green) were determined using Image J software (V 1.5). (*n*=5; average ± SEM, *p<0.05, **p<0.01).

We next investigated the functionality of exosomes from the EMT model cell lines to enhance angiogenic behaviour of endothelial cells *in vitro* and *in vivo*. Firstly, following pre-treatment with exosomes, recipient 2F-2B cell motility was assessed using transwell assay. Motility of 2F-2B cells treated with MDCK exosomes showed no change, compared to the vehicle control (no exosome supplementation), while 2F-2B cell migration was significantly enhanced by MDCK^YBX1^ or 21D1 cell exosomes (Figure 3a). Next, recipient 2F-2B tube formation was assessed, and cells stimulated with MDCK^YBX1^ and 21D1 cell exosomes exhibited increased vessel length and number of vessels (Figure 3b). Finally, we utilized matrigel plugs to examine *in vivo* angiogenic behaviour of recipient 2F-2B cells. Exosome-treated 2F-2B cells embedded in matrigel were subcutaneously injected into NOD/SCID mice, and after 21 days, tail vein injections of FITC-dextran were administered. Matrigel plugs were excised and imaged (Supplementary Figure S3). Furthermore, sectioning of plugs and fluorescence imaging revealed the absence of FITC-dextran in MDCK exosome-treated cell plugs, indicating no systemic perfusion (Figure 3c–3d). By contrast, plugs conditioned with MDCK^YBX1^ or 21D1 cell exosomes stained strongly for FITC-dextran (Figure 3c–3d), indicating presence of vessels perfused by the circulation. Together, this data demonstrates that exosomes derived from oncogenic epithelial cells undergoing EMT, but not normal epithelial cells, can promote angiogenesis in recipient endothelial cells.

### Proteomics-based identification of exosomal proteins

**Figure 4:**
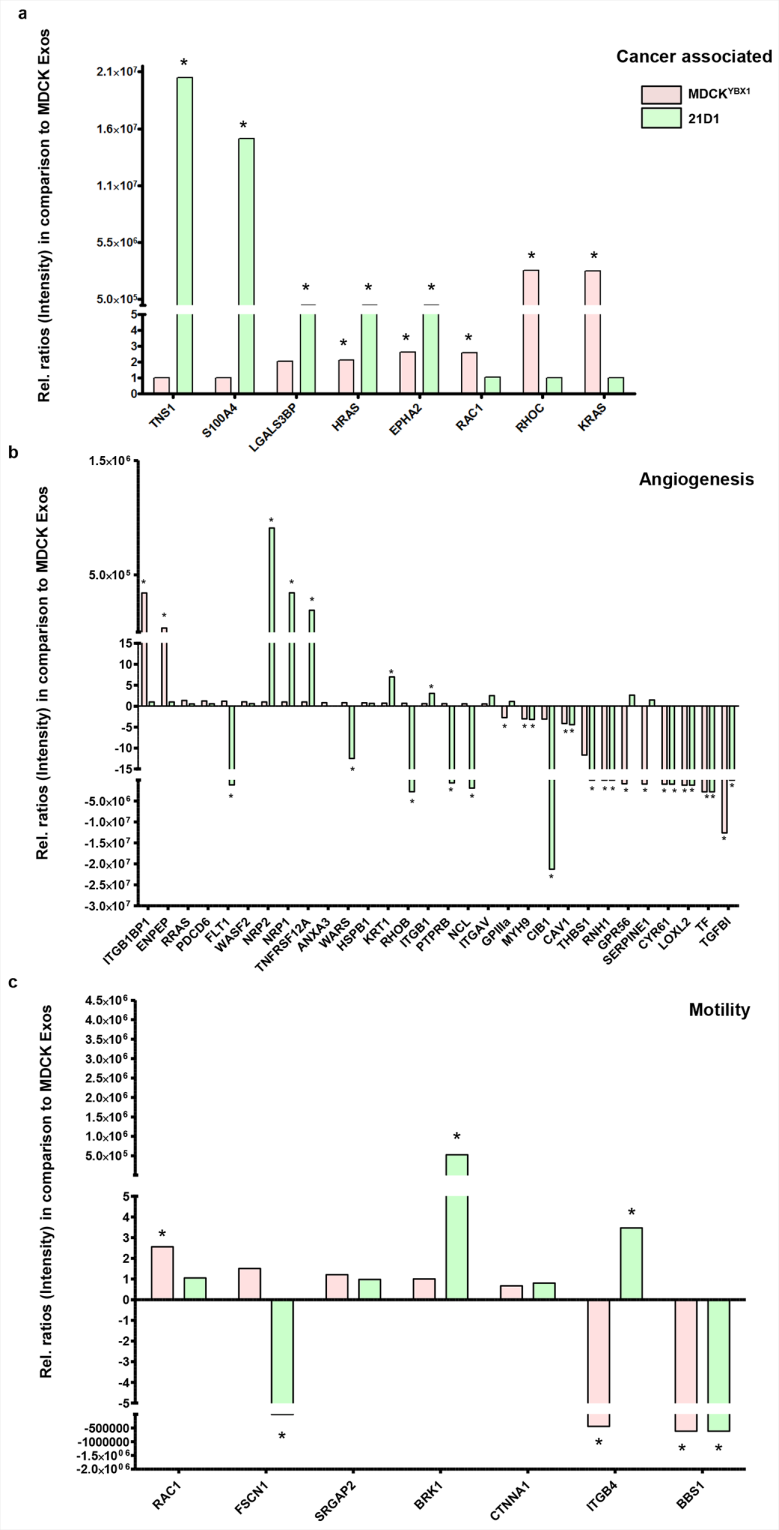
Proteomic-based quantification of factors enriched in MDCK^YBX1^ or 21D1 exosomes, compared to MDCK exosomes Exosomes were isolated from all three EMT cell lines and subjected to mass spectrometry analysis. Label-free quantification using LFQ intensity (MaxQuant) was used to reveal significant enrichment of factors associated with **a.** cancer progression, **b.** angiogenesis, and **c.** cell motility in MDCK^YBX1^ or 21D1 exosomes, relative to MDCK exosomes (*n*=2, *p<0.05).

To identify protein constituents in the exosomes involved in conferring pro-angiogenic properties, exosomes from all three cell lines were subjected to mass spectrometry analysis. As expected, the exosome protein composition showed some similarities (356 proteins), as well as unique protein characteristics (Supplementary Figure S4, and Supplementary Tables S1–S2). Given we have previously compared MDCK and 21D1 exosomes [22], we focussed on examining MDCK and MDCK^YBX1^ exosome composition. Label-free quantification revealed that 161 proteins were significantly differentially expressed (Fold change > 2 and p–value < 0.05) (Supplementary Table S2). Unbiased gene ontology mining uncovered several cancer-associated/oncogenic proteins with elevated expression in MDCK^YBX1^ exosomes, including signalling proteins (K-Ras, RhoC, Rac1, and H-Ras) (Figure 4a, Supplementary Table S3). Additionally, proteins implicated in angiogenesis including ITGB1BP1, ENPEP, were enriched in MDCK^YBX1^ exosomes (Figure 4b, Supplementary Table S3). Furthermore, examination of cell motility proteins highlighted increased abundance of Rac1 in MDCK^YBX1^ exosomes (Figure 4c, Supplementary Table S3). We overlay, quantitative comparisons of exosome proteins from MDCK and 21D1 cells, and observed many similar cancer associated proteins were also up-regulated in 21D1 exosomes (Figure 4a). However, proteins associated with angiogenesis (NRP1, NRP2 and TNFRSF12A) and cell motility (BRK1 and ITGB4) in 21D1 exosomes were very different from those proteins up-regulated in MDCK^YBX1^ exosomes (Figure 4b–4c). This suggests that 21D1 cells may utilize a different suite of molecular effectors in exosomes to promote angiogenesis, and these may be downstream of oncogenic H-Ras.

### MDCK^YBX1^ exosomes are enriched with activated Rac1

**Figure 5:**
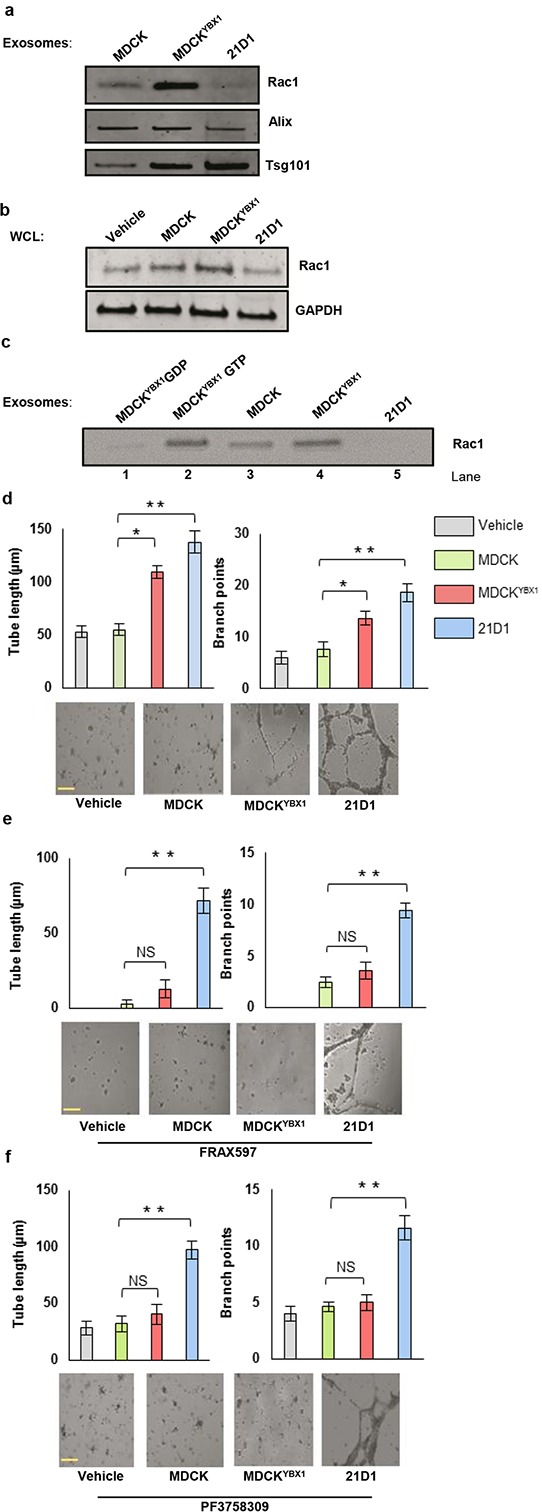
MDCK^YBX1^ exosomes contain active Rac1 and promote 2F-2B cell tube formation **a.** Expression of Rac1 in exosomes from MDCK, MDCK^YBX1^ and 21D1 cells by western immuno-blotting. **b.** Western-blot determination of Rac1 expression in 2F-2B cellular lysates following supplementation with exosomes from EMT cell lines. **c.** Detection of active Rac1 in exosomes using the Rac1 activation assay kit (Cell Biolabs). Controls were loaded with either GDP or GTP (Lanes 1-2), and active Rac1 (GTP-bound) immuno-isolated using PAK-PBD agarose beads. Rac1 was detected by western immuno-blotting. **d.** Tube formation assays using 2F-2B cells supplemented with exosomes were conducted using 1 mg/mL Matrigel, and tube length and branch points quantified. **e-f.** Tube formation assays using 2F-2B cells (7×10^4^) treated with PAK inhibitors, prior to exosome supplementation. Cells were treated with either (e) FRAX597 (1 μM) or (f) PF-3758309 (1 μM) for 1 h, and seeded onto Matrigel. 2F-2B cells were supplemented with exosomes (30 μg). After 24 h culture, tube formation was analysed and imaged. Scale bar = 50 μm. (representative images from *n*=3, *p<0.05, **p<0.01 and NS = no significant difference).

Elevated expression of Rac1 in MDCK^YBX1^ exosomes was selected for further investigation given its known association in promoting cell motility and angiogenesis. Western blotting validated increased Rac1 expression in MDCK^YBX1^ exosomes, compared to MDCK exosomes (Figure 5a). In addition, 2F-2B cells were supplemented with either MDCK, MDCK^YBX1^, or 21D1 exosomes, and blotting of cellular lysates revealed increased Rac1 levels in cells treated with MDCK^YBX1^ exosomes (Figure 5b, Supplementary Figure S5). This suggests that these exosomes containing elevated Rac1 could act as delivery vehicles to transfer cargo to recipient cells. To confirm whether Rac1 in MDCK^YBX1^ exosomes represented active Rac1, we next immuno-isolated GTP-bound Rac1 from exosomes using PAK-PBD agarose beads. As controls to demonstrate specificity of isolation, MDCK^YBX1^ exosomes were lysed, and Rac1 loaded with GDP (negative) or GTP (positive) (Figure 5c, Lanes 1-2). Simultaneously, GTP-Rac1 was isolated from MDCK, MDCK^YBX1^ and 21D1 exosome lysates, revealing highest expression in MDCK^YBX1^ exosomes (Figure 5c, Lanes 3-5, and Supplementary Figure S6). Furthermore, comparison of known Rac1 interactions in the STRING database (Supplementary Figure S7) with our proteomics dataset (Supplementary Table S1) revealed that p21-activated kinase 2 (PAK2) was also abundantly expressed in MDCK^YBX1^ exosomes. Therefore, these exosomes contain active Rac1 as well as downstream mediators that can propagate Rac1 signalling.

### Inhibition of Rac1 signalling in recipient endothelial cells inhibits angiogenesis promoted by MDCK^YBX1^ exosomes

To validate the ability of MDCK^YBX1^ exosomes to stimulate angiogenesis in recipient cells via Rac1 signaling, we inhibited downstream Rac1 targets such as PAKs in endothelial cells prior to exosome treatment. Non-PAK-inhibited 2F-2B cells show increased tube length formation and branch points following MDCK^YBX1^ and 21D1 exosome supplementation, but MDCK exosomes had no effect compared to the vehicle control (Figure 5d). However, treatment of 2F-2B cells with FRAX597, a Group I PAK inhibitor [25], reduced tube formation in MDCK^YBX1^ exosome-treated cells to baseline, but did not impede tube formation in 21D1-exosome-treated cells to the same extent (Figure 5e). We also performed the same experiment with PF-3758309, a compound that targets both Group I and II PAKs [26]. Similar to FRAX597 experiments, we observed the tube length and branch points of 2F-2B cells treated with MDCK^YBX1^ exosomes, reduced to baseline and that of cells treated with MDCK exosomes (Figure 5f), while 2F-2B cells treated with 21D1 exosomes were not altered dramatically (Figure 5f). Similar functional experiments were performed in recipient Human Umbilical Vein Endothelial Cells (HUVECs) (Supplementary Figure S8), further confirming the ability of exosomal Rac1 signalling to promote the angiogenic behaviour endothelial cells. Together, these experiments demonstrate that the angiogenic effects conferred by MDCK^YBX1^ exosomes can be blocked by inhibiting downstream PAK signalling in recipient cells.

## DISCUSSION

**Figure 6:**
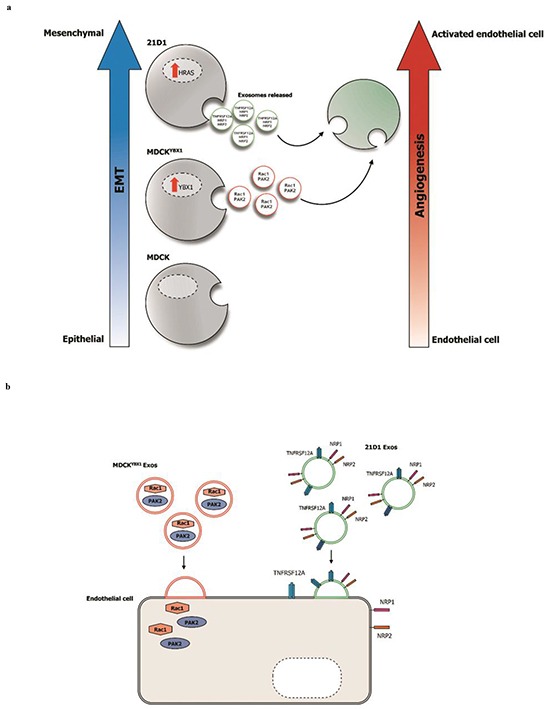
Exosomes released from oncogenic cells undergoing EMT can promote angiogenesis in recipient endothelial cells **a.** Elevated expression of YBX1 in MDCK cells induces p-EMT, while H-Ras induces a complete mesenchymal-like phenotype (21D1 cells). Exosomes derived from these cells can activate recipient endothelial cells, and enhance their angiogenic behavior. 21D1 exosomes produce a greater angiogenic response, compared to MDCK^YBX1^ cells, possibly from differential molecular constituents. **b.** Exosome cargo can be delivered to endothelial cells and influence their angiogenic behavior. Soluble proteins such as Rac1 and PAK2 may directly impact downstream cellular signaling pathways, while transfer of exosome transmembrane proteins (NRP1, NRP2, or TNFRSF12A) could alter endothelial cell surface interactions.

The TM contains various EVs and soluble protein factors that can enhance the metastatic cascade [27]. In this study, we investigated whether exosomes released from cells undergoing EMT (partial and complete) could promote angiogenesis in recipient endothelial cells. The angiogenic properties observed in 2F-2B cells were most pronounced when cells were stimulated with 21D1 exosomes, compared to MDCK^YBX1^ exosomes. This suggests that increased donor cell oncogenicity may translate into their exosomes being more potent, and lead to more heightened functional effects in recipient endothelial cells (Figure 6a). Moreover, we suspect that this property may be attributed to the molecular composition of exosomes from the donor cell (Figure 6b), and being driven by molecular perturbations in the cell (elevated YBX1 vs H-Ras).

We have previously analysed the protein constituents of 21D1 exosomes [22], and in this study focussed on molecular effectors that may promote angiogenesis. Compared to MDCK exosomes, 21D1 exosomes were enriched with angiogenesis-associated proteins NRP1, NRP2, and TNFRSF12A. Notably, all these proteins are transmembrane receptors, however, the functional significance of their identification in 21D1 exosomes is still not clear. NRP1 and NRP2 are 120-130 kDa multifunctional receptors that exist in multiple isoforms and soluble forms [28, 29]. NRP1 binds and complexes with both VEGFR-1 and VEGFR-2, and potentiates the binding of VEGF-A and activation of pro-angiogenic signalling [30, 31]. Further, NRP2 interacts as a co-receptor with VEGFR-2 and VEGFR-3 and promotes human endothelial cell survival and migration [32]. As VEGF can bind to NRP1, it is tempting to speculate that exosomal NRP1 may help present VEGF to recipient endothelial cells, or stabilise their interaction. Interestingly, we have previously published that VEGF levels were dramatically elevated in the secretome from 21D1 cells, compared to MDCK and MDCK^YBX1^ cells [23]. Therefore, it may be possible that soluble release of VEGF and exosomal NRP1 and NRP2 from 21D1 cells may work synergistically to activate endothelial cell angiogenesis. We anticipate that the PI3K and p38/MAPK pathways may be involved in transducing the signals for endothelial cell migration, survival, and vascular permeability [33].

MDCK^YBX1^ exosome protein constituents were markedly different from that of 21D1, and enriched with the Ras-related small GTPase of the Rho family, Rac1. Cellular Rac1 is involved in angiogenesis [34], and activates endothelial cell lumen and tubule formation [35, 36]. As we validated that MDCK^YBX1^ exosomes contain the highest levels of GTP-bound Rac1, we anticipate that following exosome fusion and transfer of active Rac1 to endothelial cells, downstream Rac1 signalling pathways would be activated. It is also possible that GTP-Rac1 could activate molecular effectors in the exosome prior to fusion with the recipient cell membrane. Strikingly, PAK2, a known Rac1 target, was also uniquely found in MDCK^YBX1^ exosomes. Therefore, MDCK^YBX1^ exosomes may deliver both active Rac1 and PAK2, and this could stimulate angiogenic signalling in recipient cells. The signalling pathways remained to be precisely defined, however, PAK2 is known to signal through PKC [36], and the BMK1/ERK5 pathway has very recently been shown to propagate PAK2 signalling during developmental angiogenesis, as well as mature vessel maintenance [37].

Treatment of 2F-2B and HUVEC cells with PAK inhibitors severely reduced tube length and branch points of these cells. Importantly, increased tube length stimulated by MDCK^YBX1^ exosomes was restored to baseline levels by treatment of recipient cells with PAK inhibitors prior to exosome supplementation, while the effects of 21D1 exosomes were not altered significantly. Thus, increased levels of active Rac1 and PAK2 delivered by MDCK^YBX1^ exosomes would bypass ligand-receptor interactions (VEGF-VEGFR), and enhance the angiogenic phenotype in endothelial cells via an independent downstream pathway.

Our findings imply tumour angiogenesis may be induced from stages early on in the metastatic cascade, such as epithelial cells transitioning into partial or complete mesenchymal-like phenotypes. Exosomes from cells exhibiting p-EMT (MDCK^YBX1^) were able to induce angiogenesis in recipient endothelial cells *in vitro* and *in vivo*, and the levels of induction increased with exosomes from cells further along the EMT spectrum (21D1 cells). In contrast to classical soluble angiogenic factors that most often act locally within the TM, exosomes may provide a delivery vehicle that is more resistant to degradation, and potentially have a longer range of bioactivity. Exosome fusion may deliver receptor proteins such as NRP1 and TNFRSF12A from exosomes to the surface of endothelial cells, and increase their responsiveness to soluble angiogenic ligands in the TM (Figure 6b). Concurrently, exosome fusion may also deliver soluble cargo such as cellular mediators directly into endothelial cells, and abrogate the requirement for cell surface ligand-receptor interactions (Figure 6b). Therefore, the presence of active Rac1 and PAK2 in exosomes may provide a more direct avenue to induce angiogenesis in recipient endothelial cells, and represent an early mechanism in the metastatic cascade.

## MATERIALS AND METHODS

### Cell culture

MDCK and derivative cell lines (MDCK^YBX1^ and 21D1) were generated as described previously [20, 21, 23]. Endothelial cell lines (2F-2B and HUVEC) were obtained from the American Type Culture Collection (Manassas, VA, USA). Cells were cultured in Dulbecco's Modified Eagle's Medium (DMEM) with 10% fetal bovine serum (FBS), at 37°C with 10% CO_2._

### Phase contrast microscopy

Cells were washed with DMEM and imaged on an inverted Nikon Eclipse TE300 microscope equipped with a 10× objective (Nikon Plan Fluor) using an attached 12.6 mp digital camera (Nikon DXM1200C) [23].

### Exosome isolation using OptiPrep™ density gradient medium

MDCK, MDCK^YBX1^, and 21D1 cells (50x 150 mm dishes per cell line) were cultured to 70% confluence in DMEM+10% FBS, washed three times with DMEM, and cultured in DMEM for a further 24 h. Conditioned media (CM) was collected (∼750 mL) and centrifuged (480 x *g* for 5 min, 2000 x *g* for 10 min) as described [24]. Supernatants were centrifuged at 10,000 x *g* for 30 min at 4°C, and the resulting supernatants at 100,000 x *g* for 1 h to isolate crude exosomes [22, 38, 39]. Exosomes were washed in PBS, and layered onto OptiPrep™ density gradients for ultracentrifugation at 100,000 x*g* for 18 h, as previously described [40]. Twelve individual 1 mL fractions were collected (from top of the gradient with increasing density) and each fraction diluted with 2 mL PBS. After centrifugation at 100,000 x *g* for 1 h at 4°C, supernatants were discarded and pellets washed with 1 mL PBS and resuspended in 50 ml of PBS for downstream analysis [38].

### Cryo-transmission electron microscopy (cryo-EM)

Cryo-EM imaging of purified exosome preparations was performed as previously described [22, 39].

### Scanning electron microscopy (s-EM)

s-EM analyses were performed essentially as described previously [41], with minor modifications. Briefly, exosomal preparations (∼3 μg) were analyzed on a scanning electron microscope (JEOL JSM 6340F) equipped with a cold field electron emission source. Images were acquired with 10 kV acceleration voltage, 6 mm working distance (WD), 10 fields of view, and an in-lens secondary electron detector (SEI).

### Dynamic light scattering (DLS)

DLS analyses were performed as previously described [39].

### Protein quantification and immunoblotting

Protein content was estimated by 1D-SDS-PAGE/SYPRO Ruby protein staining-based densitometry, as previously described [22]. For immunoblotting (10 μg), membranes were probed with primary antibodies [mouse anti-Rac1 (Abcam, 1:1000), mouse anti-Alix mouse (Cell Signalling, 1:1000), mouse anti-TSG101 (Cell Signalling, 1:1000), and mouse anti-GAPDH (Ambion, 1:12,000)] for 1 h in TTBS (50 mM Tris, 150 mM NaCl, 0.05% (v/v) Tween 20) followed by incubation with corresponding secondary antibodies; IRDye 800 goat anti-mouse IgG or IRDye 700 goat anti-rabbit IgG (1:15000, LI-COR Biosciences), for 1 h at RT in TTBS. Immunoblots were imaged using the Odyssey Infrared Imaging System, (v3.0, LI-COR Biosciences, Nebraska USA).

### Exosome labelling and internalisation

Exosomes (300 μg) were labelled with 1 μM DiI lipophilic dye (Invitrogen) and incubated at 37°C for 30 min. Excess dye was removed by washing with PBS, and labelled exosomes were re-isolated by OptiPrep™ density gradient ultracentrifugation (described above). Recipient 2F-2B cells (2 × 10^5^) were incubated with DiI labelled Exos (10 μg) for 2 h, fixed in 4% (v/v) formaldehyde in PBS for 10 min at RT, semi-permeabilised (0.2% (v/v) Triton X-100 in PBS), washed with wash buffer (0.1% (w/v) BSA and 0.1% (v/v) Tween-20 in PBS), and incubated with DAPI (1:5,000, Invitrogen) and/or primary antibody mouse anti-Actin (1:500, Sigma). Cells were incubated with Alexa Fluor 488-conjugated goat anti-mouse IgG (Invitrogen) at RT, and subjected to confocal microscopy using a Zeiss LSM 780 confocal microscope with 100× magnification (*n* = 3).

### Endothelial cell migration assay

For the endothelial migration assay, 2F-2B cells (2 × 10^5^) were treated with vehicle control (DMEM 0% FBS), MDCK, MDCK^YBX1^ or 21D1 exosomes (30 μg) for 2h at 37°C. Cells were pelleted at 500xg, resuspended in 100 μL of DMEM and seeded onto Transwell polycarbonate membrane cell culture inserts (8.0 μm pore size, Corning). The inserts were placed into 24-well companion plates with the bottom chamber containing DMEM (0% FBS). 2F-2B cells were left to migrate through the transwell for 24 h 37°C. Inserts were removed, cells fixed (4% (v/v) formaldehyde, 10 min), and nuclei stained with DAPI. Non-migrating cells were removed from the upper side of the inserts using cotton swabs. Migrating cells were imaged using an inverted Nikon Eclipse TE300 microscope equipped with an attached 12.6 mp digital camera (Nikon DXM1200C) (*n* = 3; average ± SEM, **P < 0.01).

### Endothelial cell tube formation assay

Endothelial cell tube formation assays were performed as previously described [42]. Briefly, 2F-2B and HUVEC (7 × 10^4^ cells/well) were re-suspended in DMEM + 5% FBS, and seeded onto growth factor-reduced BD matrigel (1 mg/ml) (96-well) for 1 h. Cells were then supplemented for 2 h with MDCK, MDCK^YBX1^ or 21D1 Exos (30 μg), or the vehicle control (DMEM). For inhibitor based tube formation assays, 2F-2B and HUVEC cells (7 × 10^4^ cells/well) were pre-treated with Rac1 inhibitors: 1 μM FRAX597 (Selleck Chem) and 1 μM PF-3758309 (Selleck Chem) for 1 h. Cells were isolated (480 x *g.* 5 min) and re-suspended in DMEM + 5% FBS, and seeded onto growth factor-reduced BD matrigel (1 mg/ml) (96-well) for 1 h. Cells were then supplemented with MDCK, MDCK^YBX1^ or 21D1 Exos (30 μg), or the vehicle control (DMEM). Tube-like structures were imaged using Nikon Eclipse TE300 microscope.

### Matrigel plug angiogenesis assay

All experiments were performed in accordance with the guidelines of La Trobe University Ethics committee. *In vivo* matrigel plug angiogenesis assays were performed as previously described [43]. 2F-2B cells (2.5 × 10^5^ cells) were treated with 30 μg of MDCK, MDCK^YBX1^, 21D1 exosomes, or vehicle control (DMEM), pre-mixed with growth factor reduced matrigel (1 mg/ml, BD Biosciences) and DMEM (0% FBS, 0.5% BSA (w/v) and 1% (v/v) Pen strep (Life Technologies), and injected subcutaneously into NOD/SCID male mice (n=8) in both inguinal regions. After 21 days, IP tail vein injections of FITC-dextran 150 kDa (Invitrogen) were administered, and animals sacrificed after 1 h. Matrigel plugs were excised, fixed in 4% paraformaldehyde, cryo-protected for 10 h in 20% (w/v) sucrose and frozen in Tissue-Tek OCT compound (Sakura Finetechnical). Fresh sections (12 μm) were prepared using a cryostat microtome (Leica CM1950), nuclei stained with To-pro-3 (1:1000), and imaged using a Zeiss LSM 780 confocal microscope.

### Proteomic analysis

Proteomic experiments were performed in duplicate as previously described [23]. Briefly, exosomes from each cell line (10 μg) were lysed in SDS sample buffer (4% (w/v) SDS, 20% (v/v) glycerol, 0.01% (v/v) bromophenol blue, 0.125 M Tris-HCl, pH 6.8), and proteins separated by SDS-PAGE, and visualized by Imperial™ Protein Stain (Invitrogen). Individual samples were excised, destained (50 mM ammonium bicarbonate/acetonitrile), reduced (10 mM DTT (Calbiochem) for 30 min), alkylated (50 mM iodoacetic acid (Fluka) for 30 min) and trypsinized (0.2 μg trypsin (Promega Sequencing Grade) for 16 h at 37°C). A nanoflow UPLC instrument (Ultimate 3000 RSLCnano, Thermo Fisher Scientific) was coupled on-line to an Orbitrap Elite mass spectrometer (Thermo Fisher Scientific) with a nanoelectrospray ion source (Thermo Fisher Scientific). Peptides were loaded (Acclaim PepMap100 C18 5 mm 100Å, Thermo Fisher Scientific) and separated (PepMapRSLC C18, 50 cm, 75 μm inner diameter, 2mm 100Å, Thermo Fisher Scientific). Details of the operation of the mass spectrometer are described previously [23].

### Database searching and protein identification

Raw data was processed using MaxQuant [44] (v1.1.1.25) and searched with Andromeda against the Uniprot Canine database comprising 28698 entries (Jul-2015) and common contaminants [45, 46]. Data was searched as described [23] with a parent tolerance of 10 ppm, fragment tolerance of 0.5 Da and minimum peptide length 7, with FDR 1% at the peptide and protein levels, and data examined with label-free quantitation (LFQ) [47]. LFQ intensity values were normalized for protein length and fold change ratios calculated. Contaminants and reverse database identifications were excluded from data analysis.

### Rac1 activation assay

Activation of Rac1 was performed according to manufacturer's instructions using Rac1 activation assay kit (Cell Biolabs) [48]. Briefly, MDCK^YBX1^ Exos (75 μg) were pre-treated for 30 min at 30°C with agitation, with 10 mM GTPγS or GDP in assay buffer (125 mM HEPES, pH 7.5, 750 mM NaCl, 5% NP-40, 50 mM MgCl_2_, 5 mM EDTA, 10% glycerol) to generate positive and negative controls. These controls and MDCK, MDCK^YBX1^, 21D1 exosome samples (75 μg) were incubated with 20 μg PAK-PBD agarose beads (Cell Biolabs) and incubated for 1 h at 4°C with gentle agitation. PAK-PBD agarose beads were pelleted by benchtop centrifugation, washed in assay buffer, resuspended in SDS sample buffer, and subjected to immunoblotting or stored at 4°C for further use.

### Statistical analysis

Student's *t*-tests were calculated using GraphPad (v5.0), with *p<0.05 and **p<0.01 considered statistically significant.

## SUPPLEMENTARY FIGURES AND TABLES









## Abbreviations

21D1: Ras-transformed MDCK cells
CM: Conditioned media
CSF-1: Colony stimulating factor 1
DMEM: Dulbecco's Modified Eagle's Medium
ECM: Extracellular matrix
EMT: Epithelial-mesenchymal transition
FBS: Fetal bovine serum
MDCK: Madin-Darby canine kidney
MMP1: Matrix metalloproteinase 1
NMWL: Nominal molecular weight limit
NRP1: Neuropilin-1
NRP2: Neuropilin-2
PAK2: p21-activated kinase 2
P-EMT: partial epithelial mesenchymal transition
TGF-β: Transforming growth factor beta
TM: Tumour microenvironment
TNFRS12A: Tumor necrosis factor receptor superfamily member 12A
VEGF: Vascular endothelial growth factor
YBX1: Y-box binding protein 1.
