# Supplementary material for: Oncogenic epithelial cell-derived exosomes containing Rac1 and PAK2 induce angiogenesis in recipient endothelial cells
Source: Oncotarget. 2016 Feb 22;7(15):19709–22. doi: 10.18632/oncotarget.7573 (PMC4991413; doi:10.18632/oncotarget.7573)
Supplement: Supplementary file 1 [file oncotarget-07-19709-s001.pdf]

SUPPLEMENTARY FIGURES AND TABLES

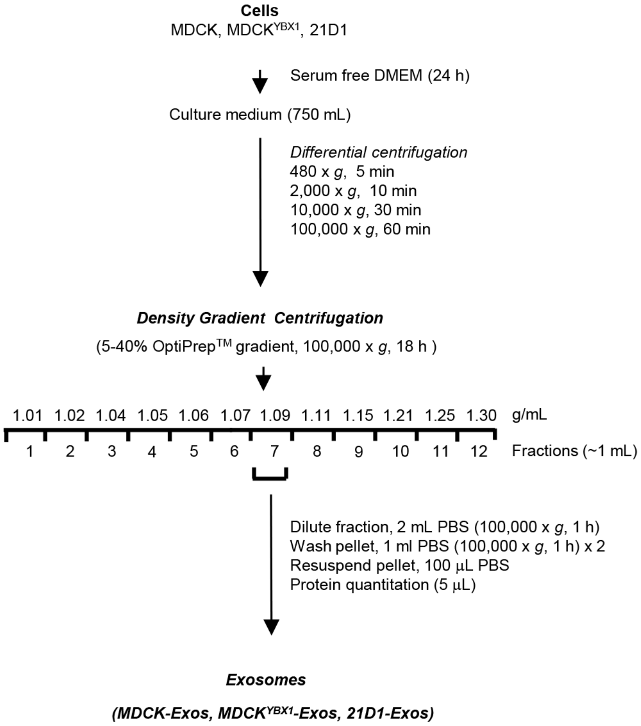

Supplementary Figure S1: Isolation of extracellular vesicles from MDCK, MDCK^{YBX1} and 21D1 cells. EMT cell lines were cultured in DMEM (containing 10% FBS), washed with DMEM (0% FBS), and cultured in serum-free DMEM for 24 h. Conditioned medium (CM) from 50 dishes of each cell line (~750 mL) was harvested and centrifuged (480 x *g*, 5 min, 2000 x *g*, 10 min) to sediment floating cells and remove cellular debris. The supernatant was then centrifuged at 10,000 x g, 30 min to remove shed microvesicles, and that supernatant ultracentrifuged at 100,000 x g, 1 h to pellet crude exosomes and extracellular vesicles. Crude vesicles were layered onto OptiPrep^{TM} density gradients, and subjected to ultracentrifugation at 100,000 x g, 18 h. 12 individual fractions were collected, and washed with PBS for downstream applications.

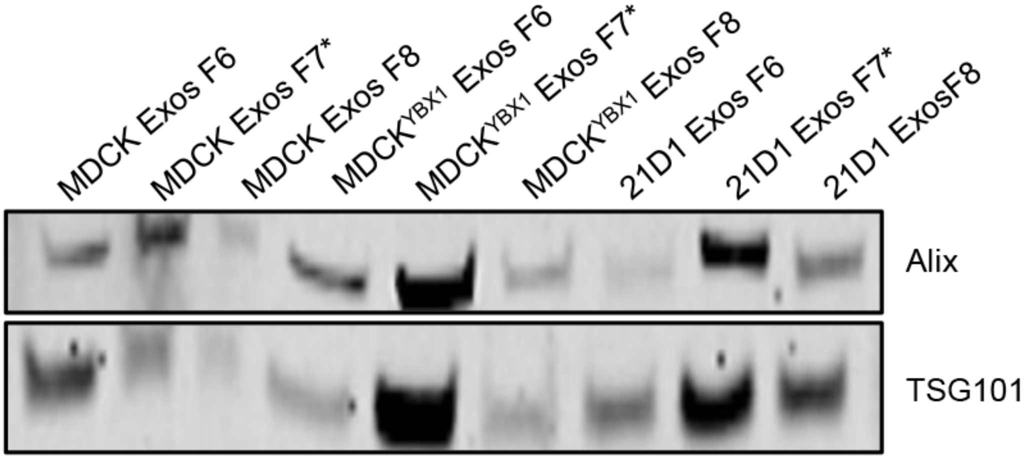

Supplementary Figure S2: Detection of exosome markers in extracellular vesicles from EMT cell lines. Expression of Alix and TSG101 in collected fractions (F6-8) by western immuno-blotting. *Central fraction F7 (1.09 g/mL) indicate greatest enrichment of Alix and TSG101.

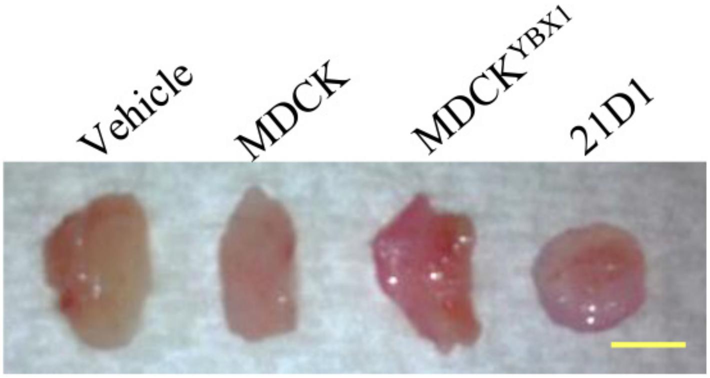

Supplementary Figure S3: Exosome-treated (with MDCK, MDCK^{YBX1} or 21D1 cell-derived exosomes) 2F-2B cells embedded in matrigel were subcutaneously injected into NOD/SCID mice, and after 21 days, tail vein injections of FITC-dextran were administered. Matrigel plugs were excised and imaged (representative images from *n*=8).

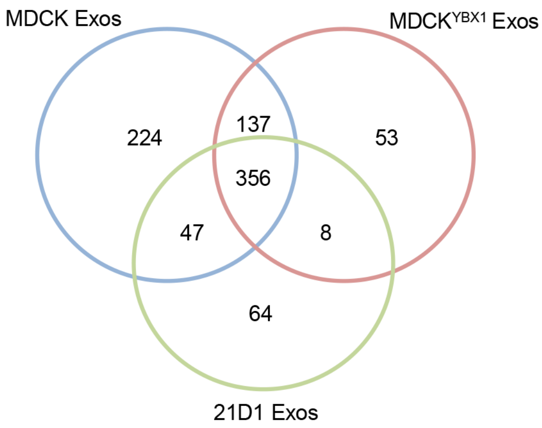

Supplementary Figure S4: Proteomic analysis of exosomes from MDCK, MDCK^{YBX1} and 21D1 cell. Comparison of proteins identified in exosomes between both replicates.

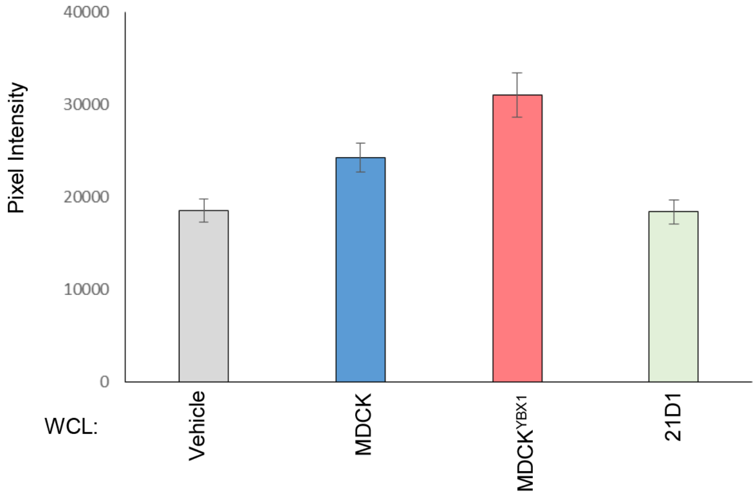

Supplementary Figure S5: Densitometry of Rac1 expression in exosome treated 2F-2B cell lysates. Comparison of Rac1 expression in 2F-2B cellular lysates following supplementation with exosomes from EMT cell lines (see Figure 5b). Pixel intensities of target bands were determined using Image Studio Lite software (V 5.2) (Licor Biosciences). (average ± SEM, *n*=3).

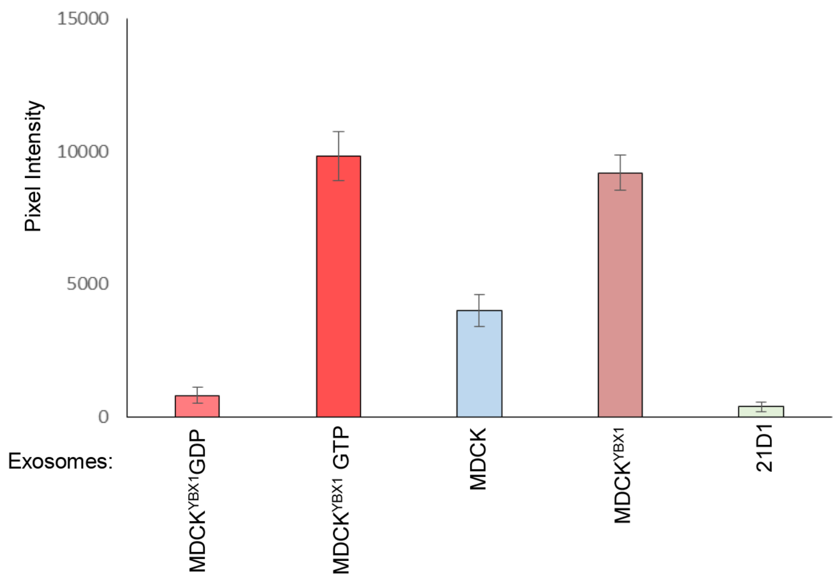

Supplementary Figure S6: Densitometric analysis of active Rac1 expression in exosomes. Comparison of active Rac1 expression by immunoblotting using Rac1 activation kit (Cell Biolabs). Controls were loaded with either GDP or GTP (Lanes 1-2), and active Rac1 (GTP-bound) immuno-isolated using PAK-PBD agarose beads. Rac1 was detected by western immuno-blotting (see Figure 5c). Pixel intensities of target bands were determined using Image Studio Lite software (V 5.2) (Licor Biosciences). (average ± SEM, *n*=3).

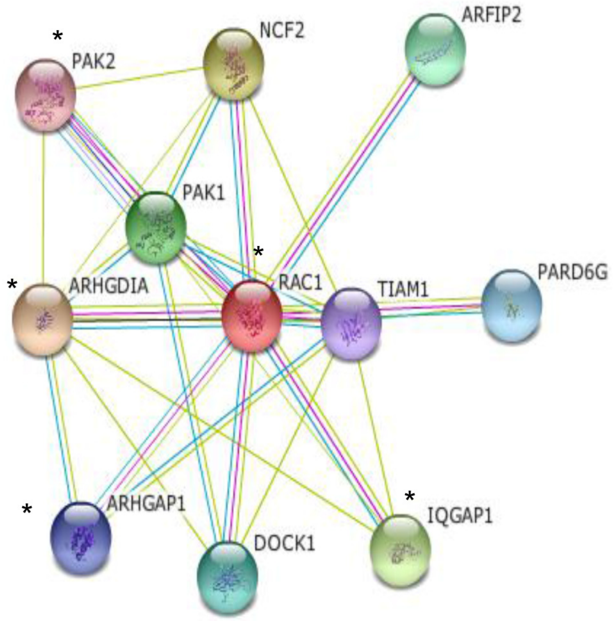

Supplementary Figure S7: Known Rac1 protein interactions. Network of proteins that interact with Rac1 in the STRING database. Components identified in this study are marked with *.

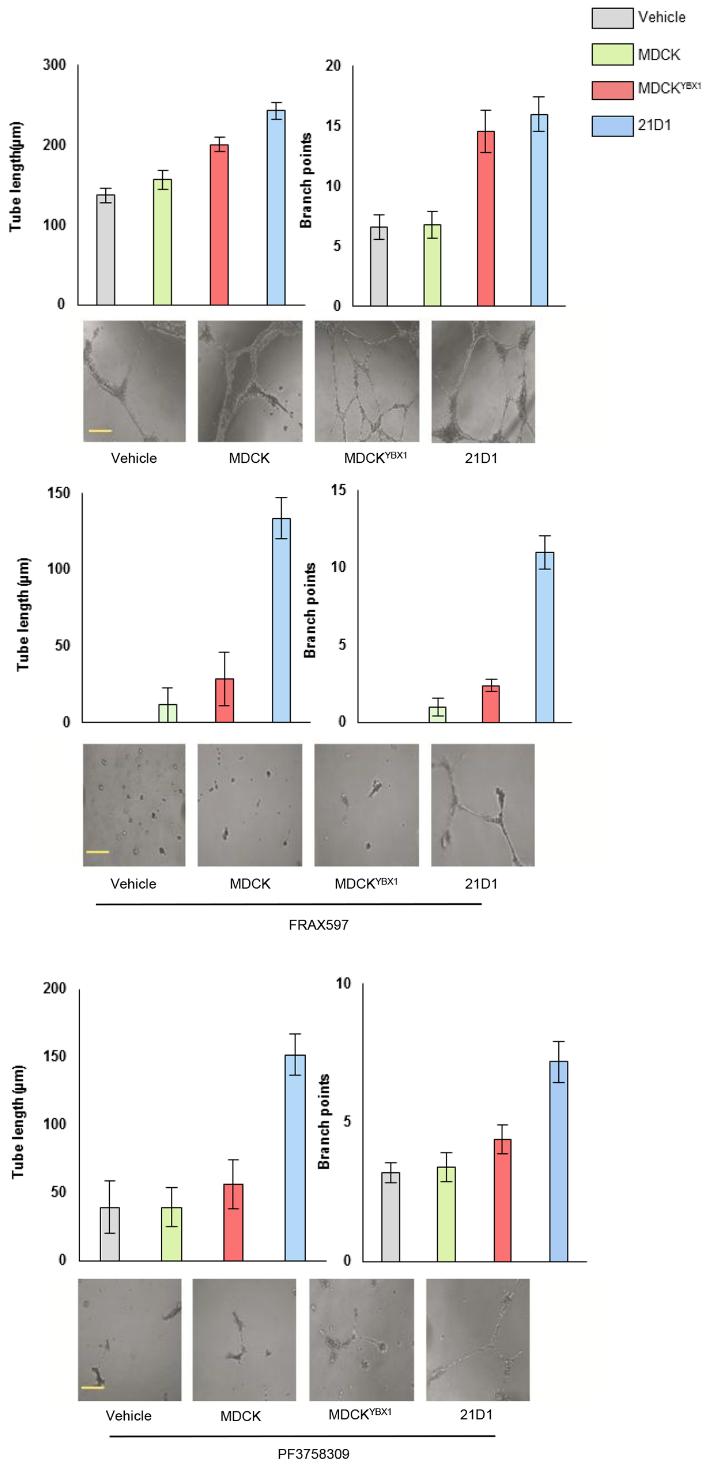

Supplementary Figure S8: PAK inhibitors limit HUVEC tube formation induced by MDCK^{YBX1} exosomes. Tube formation assays were conducted using 1 mg/mL Matrigel. HUVECs (7x10^{4}) were treated with PAK inhibitors FRAX597 (1 µM) or PF-3758309 (1 µM) for 1 h, and seeded onto Matrigel. 2F-2B cells were supplemented with exosomes (30 µg), and cultured for 24 h on Matrigel. Tube formation was analysed and imaged using inverted Nikon Eclipse TE300 microscope equipped with an attached 12.6 mp digital camera (Nikon DXM1200C). Scale bar = 50 µm (representative images from n=3).

Supplementary Table S1: Proteins identified in MDCK, MDCK^{YBX1} and 21D1 exosomes.

See Supplementary File 1

Supplementary Table S2: Proteins significantly enriched in MDCK^{YBX1}exosomes, compared to MDCK exosomes.

See Supplementary File 2

Supplementary Table S3: Relative expression of selected proteins of interest enriched in MDCK^{YBX1} exosomes.

See Supplementary File 3
